# Electroencephalogram (EEG) With or Without Transcranial Magnetic Stimulation (TMS) as Biomarkers for Post-stroke Recovery: A Narrative Review

**DOI:** 10.3389/fneur.2022.827866

**Published:** 2022-02-22

**Authors:** Zafer Keser, Samuel C. Buchl, Nathan A. Seven, Matej Markota, Heather M. Clark, David T. Jones, Giuseppe Lanzino, Robert D. Brown, Gregory A. Worrell, Brian N. Lundstrom

**Affiliations:** ^1^Department of Neurology, Mayo Clinic, Rochester, MN, United States; ^2^Department of Psychiatry, Mayo Clinic, Rochester, MN, United States; ^3^Department of Radiology, Mayo Clinic, Rochester, MN, United States; ^4^Department of Neurosurgery, Mayo Clinic, Rochester, MN, United States

**Keywords:** stroke, recovery, EEG, TMS-EEG, functional connectivity

## Abstract

Stroke is one of the leading causes of death and disability. Despite the high prevalence of stroke, characterizing the acute neural recovery patterns that follow stroke and predicting long-term recovery remains challenging. Objective methods to quantify and characterize neural injury are still lacking. Since neuroimaging methods have a poor temporal resolution, EEG has been used as a method for characterizing post-stroke recovery mechanisms for various deficits including motor, language, and cognition as well as predicting treatment response to experimental therapies. In addition, transcranial magnetic stimulation (TMS), a form of non-invasive brain stimulation, has been used in conjunction with EEG (TMS-EEG) to evaluate neurophysiology for a variety of indications. TMS-EEG has significant potential for exploring brain connectivity using focal TMS-evoked potentials and oscillations, which may allow for the system-specific delineation of recovery patterns after stroke. In this review, we summarize the use of EEG alone or in combination with TMS in post-stroke motor, language, cognition, and functional/global recovery. Overall, stroke leads to a reduction in higher frequency activity (≥8 Hz) and intra-hemispheric connectivity in the lesioned hemisphere, which creates an activity imbalance between non-lesioned and lesioned hemispheres. Compensatory activity in the non-lesioned hemisphere leads mostly to unfavorable outcomes and further aggravated interhemispheric imbalance. Balanced interhemispheric activity with increased intrahemispheric coherence in the lesioned networks correlates with improved post-stroke recovery. TMS-EEG studies reveal the clinical importance of cortical reactivity and functional connectivity within the sensorimotor cortex for motor recovery after stroke. Although post-stroke motor studies support the prognostic value of TMS-EEG, more studies are needed to determine its utility as a biomarker for recovery across domains including language, cognition, and hemispatial neglect. As a complement to MRI-based technologies, EEG-based technologies are accessible and valuable non-invasive clinical tools in stroke neurology.

## Impact Statement

EEG has been used to characterize post-stroke recovery mechanisms and to predict natural recovery and treatment response to experimental therapies. Transcranial magnetic stimulation (TMS) is a valuable add-on tool coupled with EEG (TMS-EEG) to explore effective and dynamic functional connectivity through TMS-evoked potentials (TEP) and oscillations. This review provides an in-depth summary of the use of EEG alone and combined with TMS in post-stroke motor, language, cognition, and functional/global recovery. Overall, stroke leads to a reduction in higher frequency activity (≥8 Hz) and intra-hemispheric connectivity in the lesioned hemisphere, which creates an activity imbalance between non-lesioned and lesioned hemispheres. Compensatory activity in the non-lesioned hemisphere leads to unfavorable outcomes and further aggravated interhemispheric imbalance. The main recovery mechanisms are (1) restoring interhemispheric balance (2) increasing intrahemispheric functional connectivity in the lesioned networks. TMS-EEG studies reveal the clinical importance of cortical reactivity and effective connectivity within the sensorimotor cortex for motor recovery after stroke. EEG-based technologies are particularly promising as diagnostic or predictive biomarkers for individual patients.

## Introduction

Stroke is a leading cause of functionally limiting neurological impairment and mortality in the United States, and many stroke survivors suffer from impairments of varying degrees and durations (1, 2). Predicting recovery after stroke remains a challenge (3–5). Accurately predicting post-stroke recovery at an individual level is crucial in determining the treatment needs of patients, both to improve the functional outcome for the patients and properly allocate increasingly scarce healthcare resources (6).

Electroencephalography (EEG) is a non-invasive technique that measures cortical brain activity with a high degree of temporal resolution (7). The use of high-density EEG (e.g., >60 channels) now provides a greater degree of spatial resolution (8). In addition to its clinical use in epilepsy, sleep disorders, and dementia with Lewy bodies (8, 9), EEG has been used to investigate cortical activity and functional connectivity in stroke (10, 11), consciousness disorders (12), Alzheimer's disease (13), Parkinson's disease (14), schizophrenia (15), and mood disorders (16).

In addition, EEG can be used to assess electrical responses as the brain is perturbed. Transcranial magnetic stimulation (TMS) is a non-invasive stimulation method whereby brief magnetic pulses generate focal electrical currents in the cortex. These electrical currents induce evoked potentials in focal brain areas. TMS pulses can temporarily disrupt local neural processing and help establish causal relationships between given brain areas and a person's functions (17). When used with EEG to measure the TMS-EEG evoked potentials (TEPs), TMS-EEG can identify changes to neural networks in neuropsychiatric conditions, including stroke (18).

In this review, we first summarize studies in which EEG alone was used to predict recovery after stroke. We then discuss TMS-EEG and its potential for elucidating natural recovery patterns and predicting treatment responses in stroke recovery. We conducted our literature review in Embase, PubMed, Scopus, and ScienceDirect websites with keywords; “stroke,” “recovery,” “EEG,” and/or “transcranial magnetic stimulation.” No publication date filters were applied.

## Eeg Functional and Effective Connectivity Analyses

Functional connectivity in EEG is defined as the temporal correlation of the neurophysiological activity between spatially remote areas (19). Primary functional connectivity analyses include linear and non-linear methods and information-based techniques (20). Linear brain connectivity methods use temporal cross-correlation of EEG signals in different frequency bands to estimate coherence between different brain regions (19). Techniques include power spectral analysis and bandwidth coherence (a measure based on the coupling range in time and frequency). The most common technique in stroke EEG studies is the power spectral analysis technique that quantifies coherence between spatially remote regions in different frequency band powers (often defined as delta 0.5–4 Hz, theta 4–8 Hz, alpha 8–13 Hz, beta 13–30 Hz, gamma >30 Hz). In stroke studies, delta and theta waves were defined as low frequency activity and alpha, beta, gamma as high frequency activity. As complementary techniques to linear connectivity analyses, non-linear and information-based are alternative methods (21). These techniques were not commonly used in stroke EEG studies. Therefore, we refer the reader to a detailed review by Sakkalis that contains further details on different functional connectivity techniques (20). In TMS-EEG section, we also highlighted the studies investigating effective connectivity. Effective connectivity in EEG is a metric of activity-dependent or stimulation evoked connectivity that provides a dynamic model of causal interactions between brain regions (22).

## Eeg Studies in Stroke Recovery

EEG has been used to predict post-stroke recovery (23–25), identify pathological alterations in large-scale neural networks (26), and monitor the effect of experimental therapies in different functional domains (27–29).

We review EEG studies in various behavioral systems such as motor, language, cognition, and functional/global systems since the post-stroke recovery patterns might differ by system.

### Motor System and Recovery

Table 1 summarizes the post-stroke studies of motor functioning and recovery. In most studies, bilateral sensorimotor cortices were the main region of interest, and the Fugl-Meyer Assessment (FMA) was the primary outcome measure. FMA is a comprehensive, stroke-specific assessment that quantifies and characterizes sensorimotor impairment in the affected upper and lower limbs (50). Most studies utilized power spectral analysis, event-related (motor task) oscillations, and various methods to quantify functional connectivity in neural networks. In this section, studies are summarized in four sections (1) EEG-motor performance correlation, (2) EEG as a predictor of motor recovery, (3) experimental therapies such as motor imagery (rehearsal-based therapy), brain machine interface, neurostimulation, and EEG, and (4) intense physical therapy and EEG.

**Table 1:** EEG studies of motor recovery.

**References**	***N*, Stroke patients**	**Stroke acuity at EEG collection**	**EEG data collection, timing design**	**N, EEG channels**	**Patient State during EEG recordings**	**EEG connectivity technique**	**Networks/regions investigated**	**Clinical scores collected**	**Main findings**
Giaquinto et al. (23)	34	Subacute	Longitudinal	16	Resting	PSA	Bilateral hemispheres	BI	In patients with the greatest recovery, inter-hemispheric EEG balance increased over time.
Gerloff et al. (30)	11	Chronic	Cross-Sectional	28	Behavioral-Triggered	PSA	Bilateral hemispheres, sensorimotor cortex focus	MRC, manual muscle testing	In patients who recovered well from capsular stroke, connections within motor were reduced in the stroke/lesioned hemisphere but increased in the contralesional hemisphere.
Kaiser et al. (31)	29	Subacute to chronic	Cross-sectional	61	Behavioral-triggered	ERD, ERS LC	M1s	ESS, MRC, MAS	Motor impairment correlated with contralesional ERD. Lesioned ERD correlated with spasticity. Lesioned ERS correlated with both motor impairment and spasticity.
Fallani et al. (27)	20	Subacute	Cross-sectional	61	Behavioral-triggered	SWI, imaginary coherence analysis during rest and MI	Bilateral hemispheres	FMA	Lesioned hemispheres showed a reduction in SWI scores and reduced local efficiency. Inter-hemispheric imbalance related to greater motor impairment.
Wu et al. (32)	12	Subacute to chronic	Longitudinal	256	Resting	PSA	Interhemispheric connections between M1s, lesioned connectivity in sensorimotor network	FMA-UE.	At baseline, connectivity in lesioned M1 is marker of motor status. Increase in connectivity in lesioned M1 biomarker of motor recovery. Lesioned M1–SMA connectivity increased and M1–parietal connectivity decreased in parallel with motor gains,
Bönstrup et al. (33)	12	Acute to chronic	Longitudinal	64	Behavioral-Triggered	PSA	Sensorimotor network	FMA-UE NHPT, grip strength	Initial up-regulation of brain activity after stroke correlates with neuronal reorganization for post-stroke recovery
Pichiorri et al. (28)	28	Subacute	Cross-sectional	61	Behavioral-triggered	PSA, PDC	Bilateral hemispheres	FMA-UE	Post-BCI MI training desynchronized alpha and beta activity, which correlated with motor improvement.
Thibaut et al. (34)	55	Chronic	Cross-Sectional	128	Resting	PSA	Bilateral frontal, central and parietal networks	FMA	Patients with balanced interhemispheric beta activity experienced greater motor function recovery.
Philips et al. (35)	30	Chronic	Longitudinal	58	Behavioral-Triggered	GMA and Network Based Analysis	Bilateral hemispheres	FMA-UE	Reduced contralesional intradensity and high initial values of local lesioned efficiency predicted better motor recovery.
Agius Anastasi et al. (36)	10	Subacute	Longitudinal	32	Behavioral-triggered	BSI	Bilateral hemispheres	Motricity index, FMA	Baseline BSI higher in stroke and more pronounced in the cortical stroke and predicted FMA.
Chen et al. (37)	37	Subacute	Cross-sectional	32	Behavioral-triggered	DCM, PSA	SMA and bilateral M1s	WMFT, FMA-UE,	Beta plus gamma or theta network features predicted good recovery.
Pichiorri et al. (38)	30	Subacute	Cross-sectional	64	Resting	PDC-connectivity	Sensorimotor network	TMS-CST integrity, European stroke scale and FMA	Inter-hemispheric coupling was higher in patients with preserved CST integrity. Lower sensorimotor beta coupling correlated with clinical impairment.
Vecchio et al. (39)	139	Acute	Cross-sectional	27	Resting	SWI	Bilateral hemispheres	NIHSS, BI, and ARAT	NIHSS, Barthel, and ARAT scores correlated with SWI. Baseline gamma SWI predicted final NIHSS.
Eldeeb et al. (40)	3	Chronic	Longitudinal	15	Behavioral-triggered	PDC-based network connectivity	Sensorimotor cortex	FMA-UE, grip strength.	An NIBS intervention led to improvement in PDC; improvements in PDC correlated with improvements in hand function.
Bönstrup et al. (41)	30	Chronic	Cross-sectional	64	Behavioral-triggered	PSA	Lesioned parietofrontal motor network	UEFM, NHPT, grip strength	Parietofrontal coupling was stronger in stroke patients and correlated with residual motor impairment.
Saes et al. (42)	21	Chronic	Cross-sectional	64	Resting	PSA	Bilateral hemispheres	FMA-UE	Stroke patients showed higher BSI scores between M1s, with activity differences most pronounced in delta and theta frequency bands. In the delta and theta bands, BSI negatively associated with FM-UE.
Bönstrup et al. (43)	33	Acute to subacute	Longitudinal	64	Behavioral-triggered	PSA	SMA, M1	FMA-UE, NHPT, grip strength	Acute stroke–lesioned brains failed to generate the LFO signal. LFOs progressively increased at 1 and 3 months. Re-emergence of the LFO correlated with motor recovery.
Bartur et al. (44)	14	Subacute	Cross-sectional	64	Behavioral-triggered	ERD	Bilateral M1s	FMA, BBT	Lesioned ERD positively correlated with residual motor function and the magnitude of EMG in the hand.
Cassidy et al. (45)	62	Acute, subacute, chronic	Longitudinal	256	Resting	PSA	Interhemispheric connections between M1s and intra-hemispheric motor connections	FMA-UE.	Greater coherence between inter-hemispheric delta M1 activity correlated with poorer motor status. Decreases in inter-hemispheric coherence between lesioned M1 and contralesional M1 correlated with better motor recovery.
Romagosa et al. (46)	36	Acute to subacute	Cross-sectional	16	Resting	BSI, LC	Bilateral hemispheres	FMA, BBT, NHPT, MOCA, BI.	BSI correlated with FMA-UE, but not with FMA-LE. Laterality coefficient correlated with FMA-UE and FMA-LE.
Kawano et al. (47)	40	Subacute	Cross-sectional	19	Resting	Phase synchrony index	Inter-hemispheric connections between M1s and intra-hemispheric motor connections	FMA-UE	The inter-hemispheric motor cortical alpha-band PSI was lower in stroke patients and correlated with UEFM. Contralesional central theta-band PSI was higher in patients, and correlated with improvements in FMA-UE.
Hoshino et al. (48)	24	Subacute	Cross-sectional	5	Behavioral-triggered	Amplitude envelop correlations	Frontocentral motor areas	FMA	Bilaterally higher intrahemispheric and interhemispheric activity at 4 weeks predicted higher and lower limb function at 8 weeks.
Saes et al. (49)	39	Acute	Cross-sectional	62	Resting	PSA	Bilateral hemispheres	FMA-UE	Baseline BSI theta values predicted greater upper limb motor impairment 6 months after stroke.

*ARAT, Action Research Arm Test; BI, Barthel Index; BSI, Brain Symmetry Index; Chronic, 6 months and beyond; CST, Corticospinal Tract; DCM, dynamic causal modeling; ERD, Event-related desynchronization; EMG, Electromyography; ERS, Event-related synchronization; FC, functional connectivity; FMA, Fugl Meyer Assessment; GMA, generalized measure of association. Acute; 0–2 weeks after stroke; LC, laterality coefficient; LE, Lower Extremity; BBT, Box and Block Test; LFO, Low-Frequency Brain Oscillations; M1, Primary Motor Cortex; MI, Motor imagery; NIHSS, National Institute of Health Stroke Scale; PDC, Partial Direct Coherence; PSA, Power Spectral Analysis; pts, patients; Subacute, 2 weeks-6 months after stroke; SWI, Small World index; UE, Upper Extremity*.

EEG was used to characterize neural networks for motor deficits and to correlate with motor performance. Kaiser and colleagues used high-density EEG recordings during a motor task (31). They found that event-related desynchronization of EEG waves in the contralesional motor areas and event-related synchronizations of EEG waves in the lesioned motor areas correlated with poor motor outcomes. These findings were replicated by Bartur, who showed that the magnitude of event-related desynchronization in the lesioned motor areas positively correlated with residual motor functions and EMG activity of the paretic arm (44). Dubovik et al. similarly identified specific neural networks by using alpha oscillation synchrony between various regions as a biomarker in subacute stroke (51). Interhemispheric balance and coupling between sensorimotor areas were also found to be associated with higher corticospinal tract integrity and better clinical outcomes (38). Beyond interhemispheric coupling, parietofrontal coupling was also associated with better finger dexterity in chronic stroke patients (43). Kawano et al. described another methodology termed phase synchrony index to study the resting state cortical connectivity and found that compared to controls, stroke patients with poor motor function have lower interhemispheric connectivity between motor cortices in the alpha band (47). In summary, EEG-motor performance correlation studies showed EEG could be reliably used to understand the neurophysiology of the motor system further.

EEG has also been used to predict recovery following stroke. Giaquito et al. studied low-density EEG longitudinally (3–10 recordings) in subacute middle cerebral artery stroke and showed that the initial asymmetry in high frequency activity (≥8 Hz) between hemispheres diminished over time, accompanied by improvement in motor scores (23). Similarly, better motor performance was associated with more balanced high-frequency activity between hemispheres (34, 49), especially in primary motor cortices (37, 48). Bönstrup et al. similarly showed that early transient over-activation in motor areas after stroke might play a role in post-stroke motor recovery (33). A brain symmetry index was established as a biomarker of motor functionality (42) and recovery (36), with higher scores indicating increased asymmetry in cross-cortical activity. This index was higher in acute stroke patients, especially in cortical stroke, and normalized over time with spontaneous recovery (36). The same index correlated with upper limb motor function but not lower limb function for unclear reasons (46). As another marker, low-frequency oscillations (3–5 Hz) were studied in stroke patients, and the re-emergence (43) or presence (45) of these oscillations in the lesioned hemisphere was accompanied by better motor performance and favorable recovery in various motor functions. Interestingly, contrary to other studies, it was shown that patients who recovered from stroke favorably after a capsular infarct had increased cortico-cortical connectivity in motor areas of the contralesional hemisphere, suggesting a potential compensatory mechanism for the recovery (30). The role of the contralesional hemisphere in recovery is likely not always deleterious and might vary depending upon stroke type and deficits. In sum, these studies indicate that balanced alpha and beta frequency activity between bilateral motor areas was a predictor of favorable motor recovery after stroke.

There are currently various experimental therapies that have been proven to be effective in modulating cortical neural patterns after stroke, such as those based on brain-machine interfaces (52), neuro-stimulation either with electrical stimulation (53, 54) or with transcranial magnetic stimulation (55), motor imagery training (rehearsal-based intervention) (27). These methods were tested to enhance motor recovery after stroke, and the effects of these interventional on network connectivity in stroke patients have been studied with EEG. In a group of patients with subacute stroke, Fallani et al. showed that the lesioned hemisphere exhibited reduced small-world index (a surrogate marker for density, cohesion, and integrity of local network activity) and local efficiency in connectivity during motor imagery (27). These changes were simultaneously associated with abnormally increased interhemispheric connectivity. Fugl Meyer scores were only associated with intra-hemispheric connectivity in the lesioned hemisphere, so the role of the contralesional hemisphere during motor imagery remained unclear (27). The small-world index was also used in the acute stroke period, and increased local connectivity was found to positively predict overall recovery (39). Pichiorri et a. also studied the effects of motor imagery following training with brain-computer interfaces (BCI) and observed stronger desynchronization of EEG waves in the motor areas and attributed this pattern as an underlying mechanism for motor recovery in patients with subacute stroke (28). Another study utilizing BCI based movement training in patients with chronic upper limb weakness after stroke showed a stronger desynchronization of alpha wave at the beginning of training was associated with a good motor recovery (52) In brief, EEG was able to capture the effects on functional connectivity induced by experimental therapies.

Similar to experimental therapy studies, EEG was studied before and after intensive physical therapy, which often leads to better clinical outcomes. Wu et al. investigated this phenomenon using EEG-based connectivity metrics in stroke patients undergoing upper limb intense motor therapy for 28 days (32). Reduced local connectivity in the lesioned cortex was not only a robust marker of lower scores in the FMA upper limb but also predicted good arm and hand recovery after arm training. Philips et al. also studied the effects of intensive therapy on functional connectivity by using global and local efficiency (surrogate marker for degree of cohesion of functional connectivity) metrics (35). They found that higher local efficiency in lesioned motor networks predicted favorable motor recovery. Decreases in contralesional functional connectivity and high-frequency activity (≥8 Hz) in motor areas and entire hemisphere, which led to balanced activity between hemispheres, were also associated with good motor recovery. In brief, resting and dynamic state connectivity in lesioned motor networks and balanced activity between hemispheres predicted a positive response to intensive therapies.

Although this review article focuses on studying post-stroke brain activity and its fluctuations measured by EEG, stroke and its effects go beyond the brain deficits and affect subcortical structures, such as brainstem, spine and muscles, producing imbalanced neural activations. Previous studies in healthy controls showed in the lower beta-band (14–17 Hz) activity in the bilateral sensorimotor circuits and upper beta-band (20–24 Hz) activity in the unilateral corticospinal circuits are crucial for corticospinal gain modulation (56). This potentially provides neuroscientific evidence supporting that these bands can influence cortico-muscular and/or muscular ocortical neural transmission (57).

Overall, balanced inter-hemispheric activity and increased coherence in the lesioned motor networks were found to positively predict good motor functioning and recovery. This suggests that during recovery, lesioned networks may need to increase both local and inter-hemispheric connectivity in the acute phase and then progressively become re-lateralized as behavioral performance improves.

### Language System and Recovery

Studies of post-stroke aphasia have heavily utilized power spectral analysis to calculate asymmetry indices between hemispheres (29, 58) and have mainly focused on left-hemispheric language networks. Although the type of language assessment varied across studies, most studies used language batteries that assessed the major components of language, including comprehension, fluency, and naming (see Table 2). This section organized the studies in (1) EEG predictor of language recovery in cross-sectional studies, (2) EEG in longitudinal language studies, (3) EEG as a biomarker for treatment response to language therapies.

**Table 2:** EEG language studies.

**References**	***N*, stroke patients**	**Controls**	**Stroke acuity at EEG collection**	**EEG data collection**	***N*, channels**	**State in EEG collected**	**EEG connectivity technique**	**Networks/regions investigated**	**Collected clinical scores**	**Main findings**
Jabbari et al. (65)	53	No	Acute	Cross-sectional	16	Resting	Semi quantitative analysis of slowing	Language network	PICA	Patients with pronounced slow wave activity (<8 Hz) had no or poor language recovery.
Szelies et al. (67)	23	Healthy	Subacute	Longitudinal	19	Resting	LC and PSA	Bilateral hemispheres	Token test	Low laterality coefficient in speech relevant regions of delta- and theta-band activity predicted good recovery.
Hensel et al. (25)	11	Healthy	Subacute and chronic	Longitudinal	30	Behavioral-triggered	Delta amplitude, delta dipole location and strength	Bilateral frontoparietal networks	AAT	Decreased left hemisphere delta-band activity corresponded to language recovery in the first year post-stroke, but not in the second year post-stroke.
Spironelli and Angrilli (69)	17	Healthy	Chronic	Cross-sectional	19	Behavioral-triggered	PSA	Bilateral hemispheres	AAT	Delta-band activity in language areas was higher in aphasics. Differential slow wave activity in various language tasks.
Spironelli et al. (125)	11	Healthy	Chronic	Cross-sectional	38	Behavioral-triggered	High-beta band (21–28 Hz), an index of cognitive cortical arousal	Bilateral hemispheres clustered as anterior, central, posterior	AAT	Phonological task; controls showed greater beta-band activity on the left hemisphere compared to right, whereas patients had an inverted pattern of lateralization. Reduced beta activity in perilesional areas.
Stojanovic et al. (68)	32	Healthy	Subacute	Cross-sectional	32	Resting	PSA	Bilateral frontoparietal networks	BDAE	Increased asymmetry in patients but decreased after 2 months of treatment in the subgroup of patients with good recovery.
Iyer et al. (29)	10	Healthy	Chronic	Longitudinal	128	Behavioral-triggered	Dynamic causal modeling for Event related potentials	Language Network (aMTG, pSTG, IFG, IPG, OTG)	Picture naming	Pre-treatment DCM coupling between left IPG and IFG correlated with naming improvement after treatment. Aphasics with good recovery had reduced coupling in contralateral regions post-treatment.
Dalton et al. (66)	21	Healthy	Chronic	Longitudinal	64	Resting	PSA	Bilateral hemispheres	WAB-AQ	Greater theta and lower beta in patients. Theta negatively correlated with language performance.
Kawano et al. (58)	31	Healthy	Subacute	Cross-sectional	19	Resting	Phase synchrony index (PSI)	Fronto-temporoparietal language network	Standard Language Test of Aphasia	The frontofrontal PSI was lower in aphasics and correlated positively with aphasia scores, whereas the right frontotemporal PSI was higher in aphasics and correlated negatively with aphasia scores.
^*^Nicolo et al. (70)	24	Healthy	Subacute	Longitudinal	128	Resting	FC mapping with the open-source toolbox NUTMEG	Connectivity in M1s in bilateral IFG	Geneva Bedside Aphasia Score, Nine Hole Peg, FMA-UE	Baseline beta in lesioned motor correlated with motor recovery, Beta at Broca correlated with language recovery. Global recovery associated with contralesional theta.

* *Combined language and motor systems*.

*AAT, Aachen Aphasia Test; aMTG, anterior middle temporal gyrus; BDAE, Boston Diagnostic Aphasia Examination; Chronic, 6 or more months post-stroke; CNV, Contingent Negative Variation; Acute, 0–2 weeks post-stroke; IFG, inferior frontal gyrus; IPG, inferior parietal gyrus; OTG, occipitotemporal gyrus; pSTG, posterior superior temporal gyrus; Subacute, 2 weeks-6 months post-stroke; WAB-AQ, Western Aphasia Battery-Aphasia Quotient; PICA, Porch Index of Communicative Ability*.

Language impairments and recovery after stroke were studied in various studies with a cross-sectional design. Qualitatively normal EEG was associated with good language recovery, whereas severe generalized slowing (<4 Hz) was associated with a poor recovery in an early study (65). Similarly, theta frequency activity (4–7 Hz) was more often observed in patients with post-stroke aphasia compared to healthy subjects, and it was associated with poorer performance on a measure of communication success (66). Furthermore, balanced, symmetric delta and theta activity between speech-relevant regions in the right and left hemispheres correlated with favorable language recovery (67), as did the resolution of inter-hemispheric imbalance over time (68). In another study, aphasic patients were found to have higher perilesional delta activity compared to healthy subjects, and this correlated with language impairment (69). The same group also observed topographical patterns of specific low-(<8 Hz) or high-frequency (≥8 Hz) activity that depended upon different linguistic tasks. Together, these studies delineated the functionality of several components of large-scale language networks. In brief, cross-sectional post-stroke language studies revealed increased low-frequency activity (<8 Hz) in the lesioned hemisphere, which led to inter-hemispheric imbalance, was a predictor of worse language deficits and unfavorable recovery.

In a longitudinal study, decreases in dominant left-hemispheric delta activity during the first year after stroke predicted significant language recovery but not the second year after stroke (25). Similarly, another longitudinal study revealed a correlation between favorable language and motor recovery and early subacute increases in beta activity in the lesioned motor and language areas, along with increased theta activity in contralesional homologs. However, this pattern was much less pronounced in the late subacute period (70). These studies emphasized the importance of early interventions to enhance post-stroke language recovery.

Similar to subacute studies, EEG was also studied in chronic post-stroke aphasia. Iyer et al. tested the utility of high-density EEG in a cohort of patients with chronic post-stroke aphasia undergoing 12 session-long naming therapy and used dynamic causal modeling (DCM) analysis to predict treatment response (29). DCM quantifies effective connectivity (spatiotemporal propagation of the evoked responses) between brain regions (71). Baseline functional integrity assessed via DCM coupling of the connections between left inferior parietal lobe and inferior frontal gyrus predicted an effective response to a semantic-approach naming treatment. Additionally, patients with favorable response to naming treatment overall were found to have reduced coupling in the contralesional hemisphere after therapy, suggesting the deleterious effects of the right hemisphere in picture naming recovery (29). Kawano and colleagues reported similar findings, observing increased functional connectivity, which led to an inter-hemispheric imbalance in the contralesional frontotemporal correlated negatively with aphasia scores (58).

In summary, although most post-stroke aphasia and EEG studies included relatively small sample sizes, increased alpha and beta (8 Hz and above) activity and/or preserved connectivity in the left-hemispheric language areas were predictors of favorable language functioning and recovery. Taken together, post-stroke language studies generally show that patients with more severe language impairments, poorer recovery from post-stroke aphasia, or lower treatment response to aphasia therapies were found to have increased delta and theta frequency EEG activity and reduced intra-hemispheric connectivity in the lesioned speech and language networks. Also, compensatory but ineffective activity in the right hemisphere was associated with less favorable language performance and recovery.

### Cognitive System and Recovery

Recent neuroimaging studies have allowed an analysis of the relationship between vascular-related cognitive impairment and dementia (see Table 3). In particular, MRI and PET have been studied to predict cognitive impairment after stroke (72). As an alternative or complement, neurophysiological measurements of cortical activity and connectivity through EEG can also elucidate changes in cognition following stroke and predict patient recovery (73).

**Table 3:** EEG studies of cognition.

**References**	***N*, stroke patients**	**Controls**	**Stroke acuity at EEG collection**	**EEG data collection**	***N*, channels**	**State in EEG collected**	**EEG connectivity technique**	**Networks/Regions investigated**	**Collected clinical scores**	**Main findings**
Gur et al. (24)	200	No	Acute	Cross-sectional	18	Resting	Qualitative EEG	Bilateral hemispheres	Presence of clinically diagnosed dementia	Increased slow wave activity is an indicator of subsequent cognitive decline.
Scleiger et al. (59)	20	No	Acute	Cross-sectional	8	Resting	PSA	Bifrontal	FIM with cognitive subset	Frontal DAR and global, relative alpha-band activity positively correlated with cognitive outcomes.
Song et al. (60)	105	No	Chronic	Cross-sectional	16	Resting	BRF and relative δ, θ, α, and β band power	Bilateral hemispheres	MOCA	The risk of developing cognitive impairment is 14 times higher for those with low BRF power.
Aminov et al. (61)	24	No	Acute	Cross-sectional	1	Resting	DAR and DTR	FP1	MOCA	DTR within 24 h is the predictor of MOCA scores at 90 days.
Swatridge et al. (62)	9	No	Chronic	Longitudinal	64	Behavioral-triggered	Event related potential (P300)	Bilateral hemispheres	modified Eriksen Flanker (attention and inhibition)	P300 amplitude latency is shorter after exercise (increased cortical activity). No improvement in cognitive control.
Petrovic et al. (63)	10	Healthy	Chronic	Cross-sectional	19	Resting	PSA and αAVG	Four lateral frontal, and corresponding lateral posterior	MOCA	Frontal inter-hemispheric alpha activity may be a permanent consequence of asymptomatic cognitive impairment.
Romeo et al. (64)	38	Healthy	Subacute to chronic	Cross-sectional	64	Resting	Resting state network analysis; temporal ICA for mapping in networks previously identified in fMRI	Bilateral hemispheres	FIM, attentional matrices	Tested feasibility of 10 min and map cognitive functions in resting state networks. Visuo-spatial and motor impairments were primarily associated with the dorsal attention network.
^*^Dubovik et al. (51)	20	Healthy	Subacute	Cross-sectional	128	Resting	Adaptive spatial filter and imaginary component of coherence was calculated as an index of FC.	Bilateral hemispheres	VF, VWM, SWM, NHPT, STREAM, FMA.	FC in contralesional hemisphere negatively correlated with cognitive performance. Decreases in alpha-band coherence between a given node and the rest of the brain predicted deficits, independent of anatomical lesions. Increased alpha activity in the right IFG areas correlated negatively with VF.

* *Combined motor and cognition study*.

*NHPT, Nine Hole Peg test; STREAM, Stroke; Rehabilitation Assessment of Movement; VFF, Verbal phonetic fluency; VWM, verbal working memory; SMW, spatial working memory tests; ICA, independent component analysis; fMRI, functional magnetic resonance imaging; FIM, Functional Independence Measure; BRF, Background rhythm frequency; PSA, Power Spectral Analysis; DAR, Delta alpha ratio; DTR, delta/theta ratio; MOCA, Montreal Cognitive Assessment; αAVG, the weighted average of alpha frequency; Acute, 0–2 weeks after stroke; Subacute, 2weeks-6 months after stroke; Chronic, 6 months and beyond*.

Gur et al. conducted an EEG-based qualitative assessment in a large cohort of patients with an acute stroke which showed that increased focal or global delta-theta activity (<8 Hz) could be a predictor of subsequent cognitive impairment (24). Similarly, increased delta-theta background activity (<8 Hz) activity (60, 61, 63) or reduced frontal and global alpha activity (59) were each associated with an increased risk of cognitive decline in a group of patients with acute (59, 61) and chronic (60) stroke.

Romeo et al. tested the feasibility of using high-density EEG in studying neurophysiological functional connectivity in large-scale resting-state networks defined by functional MRI (fMRI). They also correlated the functional connectivity in these networks with cognitive tests. They demonstrated that the assessment of EEG-based connectivity was broadly feasible and specifically that connectivity in the dorsal attention network correlated with visuospatial task activity (64). In a small cohort of patients with chronic stroke, Swatridge et al. studied the effects of one-time aerobic exercise on cortical reactivity and cognitive control (62). Following exercise, the P300 component of the event-related potential had a shorter latency in the central frontal lead as it suggests increased cortical reactivity as a potential marker of improvement. However, there was no improvement in cognitive control after aerobic exercise.

Notably, EEG studies to date have generally explored post-stroke cognitive impairment using cross-sectional studies of cortical neurophysiology with small sample sizes. Longitudinal data collection with larger sample sizes is needed to conclusively demonstrate the predictive ability of EEG in post-stroke cognitive recovery.

### Functional/Global Recovery

EEG-based power density maps for high (≥8 Hz)—and low-frequency (<8 Hz) bands with asymmetry indices have also been used to predict global and functional recovery and explore other domains, such as post-stroke neglect (74). Most studies used EEG in the acute setting to predict global recovery-measured by the National Institute of Health Stroke Scale (NIHSS) and functional recovery-measured by modified Rankin Scale (mRS) in the subacute and chronic stages. Critical studies are also summarized in Table 4.

**Table 4:** EEG studies for functional and global recovery.

**References**	***N*, stroke patients**	**Controls**	**Stroke acuity at EEG collection**	**EEG data collection**	***N*, channels**	**State in EEG collected**	**EEG connectivity technique**	**Networks/regions investigated**	**Collected clinical scores**	**Main findings**
Cillessen et al. (75)	55	No	Acute	Longitudinal	21	Resting	PSA	Bilateral hemispheres	mRS	Absence of slow activity predicted a good outcome
Cuspineda et al. (76)	28	No	Acute	Cross-sectional	19	Resting	AE for each frequency band and total power	Bilateral hemispheres	mRS	Alpha and theta AE were the best predictor for short-term outcome and delta AE for long-term outcome.
Sheorajpanday et al. (77)	110	No	Acute	Cross-sectional	19	Resting	PSA, DTABR, pdBSI	Bilateral hemispheres	mRS	The pdBSI and DTABR correlated with mRS at 6 months. Dependency and mortality at 6 month were independently predicted by DTABR.
Xin et al. (78)	22	No	Acute	Cross-sectional	19	Resting	PSA, BSI	Bilateral hemispheres	mRS	BSI at admission correlated with mRS at 28 days.
Su et al. (79)	162	No	Acute	Longitudinal	11	Resting	Qualitative analysis of EEG	Bilateral hemispheres	mRS	EEG grading system including low frequency (<8 Hz) activity and suppression patterns without reactivity metrics predicted poor outcome.
Lima et al. (80)	157	No	Acute	Cross-sectional	19	Resting	Qualitative analysis of EEG	Bilateral hemispheres	mRS	Presence of epileptiform activity associated with poor outcome.
Bentes et al. (81)	151	No	Acute	Longitudinal	64	Resting	PSA, DTABR, rAP	Bilateral hemispheres	mRS	High frequency activities (≥8 Hz) associated with good outcome whereas low frequency activities with poor outcome. rAP and DTABR most reliable predictors for good recovery.
Van Kaam et al. (82)	18	Healthy	Acute	Cross-sectional	21	Resting	DAR, MSC and WPLI	Bilateral hemispheres	mRS	DAR bilaterally higher in patients than in controls, MSC and WPLI in the alpha and beta frequency bands bilaterally lower in patients.
Rogers et al. (83)	16	No	Acute	Cross-sectional	1	Resting	PSA	Left frontal lead	mRS	Acute theta values associated with good outcomes, with good positive and negative predictive values
Finnigan et al. (10)	11	Healthy	Acute	Longitudinal	64	Resting	PSA, aDCS	Bilateral hemispheres	NIHSS	aDCS correlated with the 30-day NIHSSS and 15-h DWI lesion volume.
van Putten and Tavy (84)	21	No	Acute	Longitudinal	16	Resting	PSA, BSI	Bilateral hemispheres	NIHSS	cEEG feasible. BSI correlated with NIHSS.
Finnigan et al. (85)	13	No	Acute	Cross-sectional	62	Resting	DAR, rAP	Bilateral hemispheres	NIHSS	DAR and rAP at baseline correlated with 30-day NIHSS score
Sheorajpanday et al. (86)	60	No	Acute	Cross-Sectional	19	Resting	PSA, DTABR, BSI	Bilateral hemispheres	NIHSS, mRS	DTABR predicted unfavorable outcome at day 7 in lacunar strokes but not in posterior circulation syndromes
Assenza et al. (87)	42	Healthy	Acute	Cross-sectional	19	Resting	PSA	homologous MCA regions	NIHSS	Delta and theta band powers higher bilaterally in stroke and correlated with NIHSS in both hemispheres. Contralesional delta power the only valid predictor of effective recovery.
Wu et al. (88)	12	No	Acute	Cross-sectional	256	Resting	PSA	Bilateral hemispheres, sensorimotor cortex focus	NIHSS	Global delta or beta power correlated with NIHSS score. Ipsi and contralesional SMC delta and beta powers correlated positively and inversely NIHSS, respectively.
Zappasodi et al. (89)	47	Healthy	Acute	Cross-sectional	19	Resting	Global Field Power (GFP); microstate patterns	Bilateral hemispheres	NIHSS	Microstate right parieto-occipital to left fronto-central in acute phase correlated with and predicted a better effective recovery.
Fanciullacci et al. (90)	30	Healthy	Subacute	Cross-sectional	64	Resting	PSA pdBSI and DAR	Bilateral hemispheres	NIHSS	Cortical strokes have more lesioned slowing than subcortical strokes. pdBSI negatively correlated with NIHSS in both groups.

*MSC, magnitude squared coherence; WPLI, weighted phase lag index; PSA, Power spectral analysis; NIHSS, National Institute of Health Stroke Scale; mRS, modified Rankin Scale; AE, Absolute Energies; DTABR, ((delta + theta)/(alpha + beta) ratio); pdBSI, pairwise derived Brain Symmetry Index; rAP, Relative alpha power; aDCS, Acute delta change index; DAR, Delta: alpha ratio; Acute, 0–2 weeks after stroke; Subacute, 2weeks-6 months after stroke; Chronic, 6 months and beyond*.

Cross-sectional studies showed that reduced high-frequency activity (≥8 Hz) and increased low-frequency activity (<8 Hz) in the lesioned hemisphere predicted poor functional long-term outcomes as quantified by the modified Rankin Scale (mRS) (75, 76, 81, 83, 88). Brain asymmetry indices that quantify inter-hemispheric imbalance at the acute stage of recovery also reliably predicted long-term functional and global recovery (77, 78, 84, 86, 90). Increased beta activity in the contralesional motor areas, which created an inter-hemispheric imbalance, also predicted greater global impairment and worse recovery (75).

Studies investigating global recovery quantified by NIHHS showed ratios of low-to-high frequency activity, such as a delta/alpha ratio (85) or delta/theta to alpha/beta ratio (77, 86), predicted a poor global recovery. Interestingly, an increase in alpha activity in the lesioned hemisphere within the first 24 h after the stroke predicted a late improvement in the NIHSS score (10).

Some groups utilized qualitative and pattern-based analysis and concluded that certain EEG patterns also predicted poor functional recovery: epileptiform activity, abnormal low-frequency activity (<8 Hz), or generalized and burst suppression without reactivity (79, 80). Another study studied topographical maps of distinct types of patterned activity, i.e., negative or positive signal orientations in various regions in the brain. Using global field analysis, the results showed that positive signal orientations in the left occipitoparietal region and negative orientations in the right fronto-central predicted better global recovery (89). These studies emphasized the importance of identifying patterns of good recovery in addition to power density map quantifications.

Lesion location is known to affect the recovery after stroke. Following a subacute stroke, a notable discrepancy in activity levels was also observed between cortical and subcortical strokes in the lesioned and contralesional hemispheres: Patients with subcortical strokes were found to have higher alpha activity in the affected hemisphere and lower beta activity bilaterally compared to those with cortical strokes (90).

In sum, EEG obtained in the acute setting was a reliable alternative method to immediate post-stroke functioning to predict favorable functional and global recovery after stroke.

The only EEG study to date that investigated post-stroke neglect characterized the temporal-spectral evolution of event-related synchronization and desynchronization (74). The results showed that patients with neglect exhibited pathological suppression of the right hemispheric activity in preparation for an upcoming spatial task compared to patients with no neglect or healthy controls. The degree of pathological suppression correlated with poorer performance in tests of neglect.

## Eeg Combined With TMS

TMS can elicit behavioral (e.g., finger twitches) and EEG responses by depolarizing the neurons in focal regions of the brain (91). Simultaneous EEG recording during TMS offers new avenues to explore brain connectivity and recovery patterns for functional networks after stroke.

Quantifiable measurement of the behavioral responses triggered by TMS remains limited to the motor system as the primary output measure has been motor-evoked potentials (MEPs) recorded through electromyography (EMG) (92). Combining TMS with real-time EEG measurements provides an alternative immediate, direct, and quantifiable measure of the cortical activity induced by TMS that can be applied throughout the cortex and include networks of language and cognition (93). By capturing real-time cortical reactivity to TMS evoked potentials (TEPs) and TMS-related cortical oscillations, TMS-EEG allows for the characterization of dynamic and causal functional connectivity within various large-scale networks (94).

### Technical Challenges

While promising, TMS-EEG is not without technical challenges, and limitations and artifacts present significant technical challenges. One of the main artifacts in TMS-EEG sessions is induced eye blinks, and contractions of scalp muscles, especially for TMS applied to fronto-lateral brain regions (95). Offline artifact clearance can remove some artifacts from the EEG data, but this method may also remove TMS-induced changes in cortical activity (95). Hypothesis-driven approaches (such as a priori cortical activity in specific networks after TMS pulses) can be beneficial to differentiate TMS-induced responses from muscle artifacts (18).

Another common artifact is termed TMS-induced decay artifact, which arises from the effects of TMS-induced electromagnetic fields and vibrations on the EEG electrodes (96). Strategies for overcoming this artifact include using TMS-compatible EEG electrodes (96), placing foam under the TMS coil to prevent contact between the TMS coil and electrodes (97), and positioning electrode wires perpendicular to the TMS coil (98, 99). Lastly, the auditory clicking of active TMS can lead to evoked potentials in the auditory cortex, which can be limited by using headphones and playing white noise (100).

### TMS Evoked EEG Potentials (TEPs)

TEPs are characterized by positive and negative (“peak and valley”) waveforms following TMS and can measure the functional integrity of cortical structures (101). Depending on the cortical cytoarchitecture of the stimulated areas, TEPs can lead to waveform activity with different amplitude latencies for positive (P) and negative (N) deflections (102). Typically, TEPs are well-characterized for primary motor cortex as well as dorsolateral prefrontal cortex. TEPs over the primary motor cortex are N15, P30, N45, P55, N100, P180, and N280 and TEPs over dorsolateral prefrontal cortex are N40, P60, N100, and P185 (101, 103, 104). More research is needed to reliably characterize TEPs over other regions. Evoked cortical activity can be quantified in a variety of ways, including amplitude, frequency, and area under the curve and clustered by electrodes of interest or brain area (105).

Compared to measures of MEP (motor evoked potentials), measures of TEP may offer the following advantages: whereas MEPs are also affected by the brainstem, spinal cord, or even peripheral nerve pathologies (106), TEPs can in principle directly measure cortical reactivity, without being influenced by more distal components of the nervous system. TEPs can also elucidate responses below the resting motor threshold, in contrast to MEPs (103). Additionally, TEPs can study both motor and non-motor systems, while MEP measurements are limited to the motor system (107). Finally, the effects of TEPs can also be studied in distal cortical electrodes (108) while MEP measurements are restricted to the primary motor cortex.

### TMS Evoked Cortical Oscillations and Analysis of Functional Connectivity in Networks

Vascular insults can lead to pathological alterations of baseline oscillation patterns, as described earlier in this review. Like TEPs, specific oscillation patterns that follow TMS pulses can provide additional information that can be used to characterize the functionality of cortical regions in dynamic or active states (109). A TMS-evoked oscillation can be classified as an evoked oscillatory response or a total oscillatory response (110). Additional methods for quantifying TMS-evoked oscillations measure the coherence at a single electrode in a phase-locked manner across TMS trials or to measure the cross-coherence between electrodes following a single TMS trial (111).

Following a TMS pulse, signal propagation patterns between cortical regions can serve as valuable metrics for effective functional connectivity within large-scale networks (112). Compared to the resting-state connectivity in EEG, TMS-evoked functional connectivity provides a better signal-to-noise ratio and offers causal insights into the associations between different cortical regions (113). In healthy subjects and patients with epilepsy, studies have quantified TMS-evoked spatiotemporal distributions, propagation of cortical potentials, and effective connectivity (112–114).

### Combined TMS and EEG in Stroke

TMS-EEG studies are summarized in Table 5. Most studies utilize single-pulse TMS rather than other protocols such as paired- or repetitive-pulse TMS. Low-density EEG was more commonly used, and sample sizes across the studies are typically small (*n* < 20). The majority of studies targeted lesioned and contralesional primary motor cortex.

**Table 5:** TMS-EEG studies in stroke.

**References**	***N* of subjects**	***N* of channels**	**TMS-EEG type**	**Region of interest (Stim)**	**Stimulation intensity per resting MT**	**EEG connectivity technique**	**Networks/regions investigated**	**Main findings**
Cipollari et al. (115)	6	20	Single pulse	Right IFG	Subthreshold	TEP	Right Broca homolog	TEP amplitudes increase with anodal tDCS and melodic intonation therapy.
Manganotti et al. (116)	9	29	Single pulse	Left M1	Subthreshold	TEP N100 component	M1	Presence of TEP N100; predictor of good recovery.
Borich et al. (117)	10	64	Single pulse	Bilateral M1s	Suprathreshold	TEP	M1, Interhemispheric connections between M1s	Increased interhemispheric beta coherence in stroke and related to motor impairment
Gray et al. (118)	13	32	Single pulse	M1	Suprathreshold	TEP	M1	Higher amplitude and delayed latency of P30 associated with poorer hand function
Pellicciari et al. (119)	13	29	Single pulse	M1 and parietal cortex	Subthreshold	TEP, TMS evoked oscillations	Sensorimotor networks	Clinical improvement associated with increased TMS-evoked alpha oscillations, which also predicted significant motor recovery
Hordacre et al. (120)	8	64	Single pulse	M1	Threshold	TEP	M1	Higher amplitude of P30 in chronic stroke pts
Palmer et al. (121)	19	32	Single pulse	M1	Suprathreshold	TEP, TMS evoked oscillations	M1, Interhemispheric connections between M1s	Evoked interhemispheric coherence correlated negatively with upper limb function.
Tscherpel et al. (122)	28	64	Single pulse	Lesioned M1	Threshold	TEP	M1, Interhemispheric connections between M1s	Less complex, slower, and more local responses to TMS in severely affected pts.
Casula et al. (123)	19	29	Single and paired pulse	M1	Suprathreshold	TEP, TMS evoked oscillations	M1, Interhemispheric connections between M1s	Better recovery associated with balanced TEPs between hemispheres
Rolle et al. (124)	14	64	Single pulse	Bilateral M1s	Suprathreshold	TEP, TMS evoked oscillations	Sensorimotor networks	Stroke pts with higher TMS-evoked functional connectivity had better motor performance.

*M1, Primary motor cortex; TEP, Transcranial magnetic stimulation (TMS) evoked potentials (TEP); N, Negative; P, Positive; MT, motor threshold; tDCS, transcranial direct current stimulation*.

In a small cohort with post-stroke aphasia, Cipollari et al. studied TEP latency and amplitude measurements before and after anodal transcranial direct current stimulation, which is often thought to be excitatory, to the right inferior frontal gyrus. Reduced latency or increased amplitude of TEP were considered as markers of increased cortical excitability or reactivity. They showed that 3 weeks of language therapy with either active or sham stimulation increased the TEPs amplitude in the right inferior gyrus at 87 ms. In contrast, only active stimulation increased the TEP amplitude in the same region at 118 ms (115). They also observed improvements in language outcomes in both sham and active treatments, though improvements with the active treatment were significantly greater. This study suggests a potential association between increased cortical excitability and behavioral outcome after brain stimulation.

Cross-sectional post-stroke motor studies particularly focused on different components of TEP of the lesioned motor cortex. The main TEP components are P30, N45, P60, N100, P180, and N280. Hordacre and colleagues showed that chronic stroke patients had a globally larger amplitude for the P30 compared to healthy subjects (120). Likewise, significantly P30 increased amplitude and delayed latency were observed in stroke patients compared to healthy subjects, and delay in P30 was associated with poorer hand function (118). Mangonatti et al. studied the predictive utility of the N100 component of TEPs and MEPs to predict motor recovery following acute ischemic stroke and found that the ability to elicit (presence of) TEP N100 in the lesioned motor cortex alone could reliably predict a good recovery (116). Interestingly, patients with brainstem infarcts had no elicitable MEPs but normal TEPs, suggesting that TEPs are a measure of cortical reactivity that can be preserved even when the corticospinal tract is severely injured at subcortical levels. This is an important finding, as TEPs are specific markers of cortical reactivity. In contrast, MEP measurements can also be affected by non-cortical structures such as the spinal cord and brainstem even peripheral nerves, as previously mentioned.

Interhemispheric connections and balance were also investigated with TMS-EEG motor studies. In a study in which interhemispheric functional connectivity in EEG was not apparent at rest, TEPs were used to unmask interhemispheric functional connectivity in chronic stroke patients (117). Casula et al. observed that single-pulse TMS pulses on contralesional created a significant interhemispheric imbalance, which led to suppression of the activity in the lesioned hemisphere (123). Furthermore, patients who later experienced a better recovery showed more balanced TMS-evoked activity between hemispheres. In post-stroke patients, Palmer and colleagues also investigated TMS-evoked interhemispheric beta coherence during rest and active muscle contraction (121). They showed single-pulse TMS on the lesioned hemisphere during an active motor task failed to evoke intrahemispheric connections. The evoked interhemispheric coherence by TMS on the lesioned hemisphere during the active task also correlated negatively with upper limb functions. Stroke-induced changes to interhemispheric interactions are an active area of research. Although the role of the contralesional hemisphere in stroke recovery remains controversial, TMS-EEG studies reliably revealed ineffective compensatory activity of the contralesional hemisphere activity during motor system recovery. The combination of TMS and EEG offers a new degree of fidelity in characterizing the interactions between hemispheres and elucidating their significance to post-stroke motor recovery.

TMS-evoked alpha oscillations in different EEG bands are used as markers for effective connectivity. In addition to cross-sectional studies, Pellicciari et al. utilized a robust longitudinal design and measured the evolution of TMS-evoked activity over days in patients with motor impairments arising from subacute stroke (119). Participants underwent TMS-EEG 20, 40, 60, and 180 days post-stroke, and those with clinical improvement were found to have increased TMS-evoked oscillations. TMS-evoked alpha oscillations at baseline also predicted significant motor recovery at 40 and 60 days. In another longitudinal study, Tscherpel et al. investigated brain responsivity as a potential biomarker for motor recovery after stroke (122). Compared to healthy controls and minimally affected patients, severely affected patients were found to have less complex, slower, and more local responses to TMS. This pattern reliably predicted motor recovery at 3 months. Also, in patients with no MEPs, TEP additionally predicted good motor recovery in a subgroup of patients.

The TMS-evoked activity was also studied in a state-dependent fashion—at rest and during an active motor task—in post-stroke patients and healthy controls (124). When analyzed as a group, healthy controls showed an increase in TMS-evoked functional connectivity during the motor task compared to rest, while stroke patients did not. Stroke patients with higher resting TMS-evoked functional connectivity performed better on the Fugl Meyer upper limb assessment.

Most TMS-EEG studies in the post-stroke period that we identified have focused on the motor system. These studies offered valuable insight into how the functionality of motor networks after the vascular insult. Higher cortical reactivity with faster and more complex evoked oscillatory activity and increased evoked functional connectivity in the lesioned motor areas reliably predicted favorable motor recovery after stroke. Studies investigating post-stroke language, cognition, neglect, and swallowing impairments are additionally needed to understand better the utility of TMS-EEG as a biomarker of recovery across multiple domains.

## Discussion

We have reviewed key studies in the stroke literature that either utilized EEG alone or in combination with TMS to delineate pathological alterations in neurophysiology. These studies focused on TMS-induced activity changes in the lesioned and contralesional hemispheres, and analyses tended to relate significant changes to post-stroke recovery across different functional domains. As a complement to MRI-based technologies, EEG-based technologies are particularly promising as diagnostic or predictive biomarkers for individual stroke patients. The bihemispheric power spectral analysis has been the most reliable analysis technique in stroke EEG studies. Although the data for functional and effective connectivity measures seem promising, given the heterogeneity of the analysis methods and small sample in the studies, more research is needed to identify reliable biomarkers that can be used widespread.

It is crucial to utilize normative data to understand neurophysiological alterations in both hemispheres after stroke. Recent studies have included age-matched healthy controls and provided further insight into stroke-induced changes (39, 42, 43). Notably, these studies revealed that substantial pathological alterations occur in the ipsilesional hemisphere as well as in the contralesional hemisphere when compared to healthy age-matched controls (58). This suggests that despite its relative distance from the focus of stroke insult, the contralesional hemisphere cannot necessarily be considered “healthy” or “unaffected.”

Recent studies have also begun to utilize high-density EEG (39, 40, 64). Although several studies have demonstrated that low density (85) and single-electrode designs (61, 83) provide valuable information in predicting recovery for global functions, as assessed by the mRS or total Montreal Cognitive Scale (MOCA) scores, high-density EEG increases spatial resolution and can lead to more detailed characterization of complex networks.

Additionally, there has been a trend in stroke research toward collecting longer-term longitudinal data such as at least a year after stroke to characterize the temporal evolution of cortical activity following stroke (45, 119). Some studies have shown that certain EEG patterns are only significant and diagnostic in specific time windows (70, 119), indicating the importance of longitudinal data collection.

EEG alone (34, 47, 48) or TMS-EEG (118, 120) have focused primarily on motor system networks/recovery or utilized global scales such as the modified Rankin Scale and National Institute of Health Stroke Scale, where scores of the motor system contribute extensively to the total score. Unlike studies of MEPs or visual or auditory evoked potentials, TMS-EEG paradigms enable the analysis of functional systems such as cognition and language without requiring sensory input or motor output measures. As cognitive and language impairments are also common after stroke (2, 72), well-designed studies are also needed in these areas to inform new treatment strategies such as neuromodulation and pharmacotherapeutics, increase the efficacy of existing behavioral therapies, and illuminate the underlying neural networks of the recovering post-stroke brain.

In conclusion, post-stroke EEG studies showed a reduction in intrahemispheric functional connectivity in various networks of the lesioned hemisphere after stroke, and this pattern was associated with poor functioning. Studies also revealed more pronounced low-frequency activity (delta or theta frequency activity) in the lesioned hemisphere and higher frequency activity (alpha or beta activity) in the contralesional hemisphere, which created an interhemispheric imbalance. A higher interhemispheric imbalance was associated with poor function, and restoration of the balance was associated with improved recovery after stroke. Other good recovery patterns included an increase in local intrahemispheric functional network connectivity and high-frequency activity (≥8 Hz) in the lesioned hemisphere. TMS-EEG studies revealed that increased cortical reactivity to TMS pulses and a higher evoked functional connectivity in the lesioned cortical networks were associated with improved recovery and function.

## Author Contributions

ZK, SB, and NS conducted the literature review, created the tables, and wrote the first draft of the manuscript. BL contributed to the conception and design of the study. All authors contributed to manuscript revision, read, and approved the submitted version.

## Funding

BL was supported by NIH NINDS K23NS112339.

## Conflict of Interest

BL has no personal financial interests; other conflicts include named inventor (Cadence Neuroscience Inc., waived rights), investigator (Medtronic EPAS, NeuroPace RESPONSE, Neuroelectrics tDCS for Epilepsy), and consultant (Epiminder, Medtronic, Philips Neuro). GW has licensed intellectual property to Cadence Neuroscience and NeuroOne Inc., and is an investigator on projects with Medtronic, NeuroOne Inc., Cadence Neuroscience, and UNEEG Inc. The remaining authors declare that the research was conducted in the absence of any commercial or financial relationships that could be construed as a potential conflict of interest.

## Publisher's Note

All claims expressed in this article are solely those of the authors and do not necessarily represent those of their affiliated organizations, or those of the publisher, the editors and the reviewers. Any product that may be evaluated in this article, or claim that may be made by its manufacturer, is not guaranteed or endorsed by the publisher.
